# Should we screen for intracranial aneurysms (IAs) in systemic hypertension at the time of cardiac magnetic resonance (CMR)?

**DOI:** 10.1186/1532-429X-17-S1-P411

**Published:** 2015-02-03

**Authors:** Jonathan C Rodrigues, Neelam Hassan, Mandy Williams, Amy E Burchell, Laura E Ratcliffe, Mark Hamilton, Emma C Hart, Angus K Nightingale, Julian F Paton, Nathan E Manghat

**Affiliations:** 1 CMR Unit, NIHR Cardiovascular Biomedical Research Unit, Bristol Heart Institute, Bristol, UK; 2 School of Physiology and Pharmacology, The University of Bristol, Bristol, UK; 3 Foundation School, Severn Postgraduate Deanery, Bristol, UK; 4 Department of Radiology, Bristol Royal Infirmary, Bristol, UK; 5 Cardionomics Research Group, Bristol Heart Institute, Bristol, UK

## Background

IAs are detected in 2.3% of adults. Systemic hypertension is a risk factor. Screening for IA in certain high-risk groups, such as patients with coarctation of the aorta, has been discussed in international guidelines. Patients with coarctation often have concomitant systemic hypertension. Moreover, the prevalence of hypertension in patients with coarctation and IA is significantly higher than those without IA. It is uncertain whether hypertension alone represents a sufficient risk factor to prompt screening for IAs.

## Methods

Consecutive patients referred from our tertiary hypertension clinic underwent comprehensive magnetic resonance assessment including CMR and 3D time-of-flight MRA imaging at 1.5T. The study was conducted in accordance with The Governance Arrangements for Research Ethics Committees. Cerebral MRAs were double reported by a blinded Neuroradiologist. Demographic data, including presentation office systolic (SBP) and diastolic blood pressures (DBP), aneurysm data and CMR-derived left ventricular mass (LVM) indexed to body surface area, age and gender were recorded. Continuous variables were compared by Student *t* tests and categoric variables by Fisher exact test (*p*<0.05 = significant).

## Results

One hundred and twenty one (n=121) MRAs were included (52% male, mean age 52±14.6 years). IAs were detected in 10 patients (8.2%) (table 1), significantly more than expected in the general population on the basis of 2% prevalence (p<0.05) and similar to the coarctation population (10.3%). Mean aneurysm size was 2.1±0.7mm (range 1-4mm). Subgroup analysis demonstrated no significant differences between those without IA (n = 111) and those with IA (n=10) by age (52.0±14.2 vs 52.3±19.7 years, p=0.9463) or gender (53% vs 40% male, p=0.5176). No difference in prevalence of hypertension subtype demonstrated between those without IA and those with IA (resistant: 45% vs 30%, p = 0.5103, difficult to treat: 12.6% vs 20%, p=0.3369, drug intolerant: 14.4% vs 30%, p=0.1913, young-onset: 18.9% vs 10%, p=0.6874, medication-controlled: 9% vs 10%, p=0.999). Inferred hypertension severity and chronicity was similar between those without IA and with IA (SBP: 171.9±28.6 vs 158.8±35.5 mmHg, p=0.1768, DBP: 97.7±14.9 vs 91.4±16.8 mmHg, p=0.2066 and indexed LV mass: 88.2±24.8 vs 87.6±25.5 g.m^-2^ p=0.9375, number of antihypertensive medications: 3.0±2.0 vs 2.6±1.6 p=0.5015).

**Table 1:** Details of aneurysm and patient demographics.

Site of aneurysm	Size (mm)	Genger	Age (years)
Right MCA	1-2	Male	47
Right ICA bifurcation	2	Female	52
Right ACA	2	Male	58
Left PCom	2	Female	74
Right distal ICA	4	Female	81
Basilar tip	2	Female	45
Left MCA	2	Female	75
Left MCA	2	Male	39
Right SCA	2	Male	26
Basilar tip	2	Female	26

MCA = middle cerebral artery, ICA = internal carotid artery, ACA = anterior communicating artery, PCom = posterior communicating artery, SCA = superior cerebellar artery, PCA = posterior cerebellar.

## Conclusions

The prevalence of IA in this cohort is significantly higher than the general population. Subgroup analysis failed to identify particularly high-risk groups within the hypertension cohort studied. All aneurysms detected were small and managed conservatively, consequently the clinical benefit of routine screening for IAs at the time of CMR in hypertensive patients remains unanswered.

## Funding

NIHR Cardiovascular Biomedical Research Unit, Bristol Heart Institute.

JCLR: Clinical Society of Bath Postgraduate Research Bursary.

ECH: BHF grant IBSRF FS/11/1/28400.

